# Observations on Medical Education

**Published:** 1813-04

**Authors:** 


*To the Editors of the Medical and Physical Journal.*

GENTLEMEN,

THE letter signed Senex, in your last Journal, has been the subject of much comment among us pupils, and many of us feel the truth of the old gentleman's remarks.

In this age of reform, if the governors or the house-committees of the two great hospitals of St. Bartholomew's and St. Thomas's, were to be awakened to a sense of the great trust committed to them, and were to read over the charge given at their appointment, they would find it as much their duty to inquire into the careful attendance of the medical officers and their pupils, as into the value of the receipts and expenditure of those noble charities. The rules already established to regulate the proceedings of these persons, need neither alteration nor amendment, they require only to be enforced. We should not then find 1000*l.* a-year expended in the article of Port Wine at one of these charities, whilst 14*l.* on an average, suffices at the other. If 1400*l.* per annum is sufficient for the medicaments of one of these establishments, why should we hear of 4500*l.* being squandered in the other? Surely there must be some sad mismanagement in these matters, and it is fit the governors should be able to explain it.

The salary of the doctors, at the house of expense, has been lately raised to 100*l.* a-year. I do not mean to object to this but; I would infer, that, at the other house, when such praiseworthy economy is observed, the doctors deserve an adequate premium for their care. Certainly, the attention to the poor patients is neither what it ought to be, nor instructive enough to render that service to the pupil which it might do; for the majority of surgeons' patients are left to the nurses, or to themselves, for the dressing of their wounds. Patients sometimes are lost by hæmorrhages and accidents, and are left to the care of youths totally uninstructed, as Senex observes, in any of the accomplishments of practical surgery.

At public schools, when the masters retire, order is kept up by the rank of monitors; and some authority similar to that is necessary for the decent conduct of such large classes. Those among us who have distinguished themselves by their regular attention and their abilities, should be presented to the treasurers, and receive from them some distinguished authority to act in the capacity of house-surgeons *pro tempore;* an appointment most necessary at St. Thomas's, being the only hospital where such an useful assistant is not allowed.

allowed. At the Westminster, London, Middlesex, and St. George's, infirmaries, a house surgeon and assistant surgeons are appointed; even at St. Bartholomew's they have long been thought necessary; but, at St. Thomas's and Guy's, the revenues of the hospitals seem the chief care of the governors.

It is indeed essential that the revenue should be carefully looked after, because one of those hospitals is giving way in so many places, that it is probable it must ere long be quite rebuilt; but, as the purpose of the charity is to benefit the poor patients, it is more essential that the conduct of those who are appointed to look after them should sometimes be inquired into. I could enlarge upon this subject, and point out the true reasons why our medical students have so little practical skill; why we have so many unlearned doctors, bungling surgeons, and clumsy men-midwives; why the company of apothecaries complain; and why the truss-makers, tooth-drawers, and oculists, ride in their chariots, and smile at the colleges. Every branch of the medical art seems to be going wrong, and each complains of the slovenly practice of its class.

A petition was lately presented by the men-midwives, praying, that their fraternity might be obliged to undergo an examination, because of the mischiefs which were done. The apothecaries now want better educated journeymen, and deeper phials; quack doctors are admitted as pupils to lectures and infirmaries; and veterinary surgeons are called into consultation.

If you think these subjects worthy your consideration, you shall hear further from

Your humble Servant,

A STUDENT.

